# Effects of microclimatic variables on the symptoms and signs onset of *Moniliophthora roreri*, causal agent of Moniliophthora pod rot in cacao

**DOI:** 10.1371/journal.pone.0184638

**Published:** 2017-10-03

**Authors:** Mariela E. Leandro-Muñoz, Philippe Tixier, Amandine Germon, Veromanitra Rakotobe, Wilbert Phillips-Mora, Siela Maximova, Jacques Avelino

**Affiliations:** 1 Agroforestry and Sustainable Agriculture Department, Division of Research and Development, Tropical Agricultural Research and Higher Education Center, Turrialba, Cartago, Costa Rica; 2 CIRAD, UPR GECO, Montpellier, France; 3 ENSAIA, Vandoeuvre, France; 4 CIRAD, UPR Bioagresseurs, Montpellier, France; 5 AgroSup Dijon, DIJON, France; 6 The Department of Plant Science, The Pennsylvania State University, University Park, Pennsylvania, United States of America; 7 IICA-PROMECAFE, Guatemala, Guatemala; Universita degli Studi di Pisa, ITALY

## Abstract

Moniliophthora Pod Rot (MPR) caused by the fungus *Moniliophthora roreri* (Cif.) Evans *et al*., is one of the main limiting factors of cocoa production in Latin America. Currently insufficient information on the biology and epidemiology of the pathogen limits the development of efficient management options to control MPR. This research aims to elucidate MPR development through the following daily microclimatic variables: minimum and maximum temperatures, wetness frequency, average temperature and relative humidity in the highly susceptible cacao clone Pound-7 (incidence = 86% 2008–2013 average). A total of 55 cohorts totaling 2,268 pods of 3–10 cm length, one to two months of age, were tagged weekly. Pods were assessed throughout their lifetime, every one or two weeks, and classified in 3 different categories: healthy, diseased with no sporulation, diseased with sporulating lesions. As a first step, we used Generalized Linear Mixed Models (GLMM) to determine with no *a priori* the period (when and for how long) each climatic variable was better related with the appearance of symptoms and sporulation. Then the significance of the candidate variables was tested in a complete GLMM. Daily average wetness frequency from day 14 to day 1, before tagging, and daily average maximum temperature from day 4 to day 21, after tagging, were the most explanatory variables of the symptoms appearance. The former was positively linked with the symptoms appearance when the latter exhibited a maximum at 30°C. The most important variables influencing sporulation were daily average minimum temperature from day 35 to day 58 and daily average maximum temperature from day 37 to day 48, both after tagging. Minimum temperature was negatively linked with the sporulation while maximum temperature was positively linked. Results indicated that the fungal microclimatic requirements vary from the early to the late cycle stages, possibly due to the pathogen’s long latent period. This information is valuable for development of new conceptual models for MPR and improvement of control methods.

## Introduction

Cacao (*Theobroma cacao*) diseases are the main threat for production, causing losses estimated at 30 to 40% worldwide [1]. Cacao has proved to be highly susceptible to new-encounter diseases as well as pests [2]. At the beginning of the 20th century, more than 50% of global cocoa production (approximately 29 400 tons) occurred in mainland tropical America, followed by the Caribbean islands, Africa, Asia and Oceania[3]. However, diseases such as Moniliophthora Pod Rot (MPR) and Witches’ Broom caused a decline in American cocoa production. Today, only 16% of world production comes from America, which represents 618 000 tons of cocoa [3]. MPR has been reported as the most destructive, invasive and difficult-to-control cacao disease in the area [4, 5]. With its advance, the disease has swept many plantations in countries where it has been reported.

MPR is caused by the fungus *Moniliophthora roreri* (Cif.) Evans *et al*, (Basidiomycete, Marasmiaceae). Its center of origin is located in Colombia [6], and from there the pathogen has spread to 12 countries in tropical America [7]: Ecuador (formerly considered the place of origin) [8, 9], Venezuela, Panama, Costa Rica, Nicaragua, Peru, Honduras, El Salvador, Guatemala, Belize [10], Mexico [11] and recently Bolivia [12]. This disease is considered a threat for cacao production since its causal agent is in a very intense invasive stage. It stayed confined to South America for decades, but in less than 30 years, it invaded Central America and got to Mexico. Apparently, most of the commercial cacao genotypes established in the region, and in the world, are susceptible. The arrival of this pathogen to countries such the Dominican Republic and Brazil, and especially to other continents, particularly to Africa would be devastating [13]. West Africa is the world’s largest cacao-producing region.

Little is known about the biology of the fungus. According to the description made by Evans, Stalpers [14] *Moniliophthora roreri* has partitioned mycelium (septa) with dolipores and without clamp connections. Hyphae are hyaline and thin-walled. Spores have basipetal formation in simple chains of four to10 branched units [15]. These features classified this fungus as basidiomycetes [14]. The spores are easily removable, with thick walls, pale yellow in color when immature or dark brown at a mature stage. They could be globose, elliptical or amorphous [6]. The characteristics of these structures vary a little depending on strains.

It is well-described that cocoa pods are susceptible to infection by *M*. *roreri* [16] and some authors have suggested that flowers could be also attacked [17, 18]. *Moniliophthora roreri* is considered a hemibiotrophic fungus and its life cycle is completed in two phases: 1) a biotrophic phase, from the germination of the spores to the intercellular invasion of the pod and 2) a necrotic phase causing growth reduction of the pods and finishing with the invasion of the fungus to the cells causing the appearance of internal and external necrosis [15].

**Fig 1:**
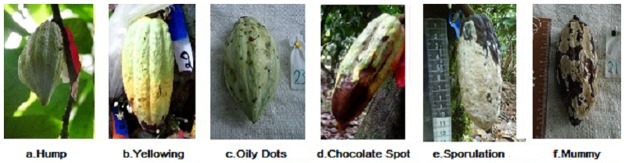
*M*. *roreri* symptoms and signs. Photos by Mariela E. Leandro-Muñoz.

This pathogen has an extensive incubation and latency period [19]. Once the fruit is infected, it may take 40 to 60 days to show external symptoms [20]. During the early infection stages, the fungus penetrates the pods intercellularly. Once inside, it invades intracellularly, destroying the fruit’s internal tissue [18, 21]. Subsequently, external symptoms appear, which may occur throughout the pod development [18]. Sometimes fruits with no visible symptoms can have a hidden infection and be rotten inside. Generally, these pods are heavier than healthy fruits since water starts to accumulate inside. When cells are destroyed, they stop working correctly their internal content is spread around and transpiration is interrupted [9, 22, 23]. Infection normally occurs in the early stages of fruit growth, and as the organ grows, it becomes more resistant [24]. One possible explanation of the resistance of mature pods is that they are simply harvested before the symptoms become obvious [25]. If pods are less than three months old, the first symptom to appear is a lump, hump or swelling (Fig 1a). If the infection occurs later, the first symptom will be the appearance of small yellow spots on older green pods and orange spots on the red ones. This symptom is also known as yellowing or early ripening (Fig 1b). Appearance of oily or aqueous spots is another early symptom (Fig 1c), followed by an irregular brown spot with a yellow halo. This symptom is known as chocolate spot (Fig 1d) [20]. Under warm and humid conditions, pathogen development is observed as a hard white stroma (mycelium) over the chocolate spot. Spores are formed over the mycelium and appear as a creamy or brown mass (Fig 1e). Lesions of infected pods that remain attached to trees can sporulate for up to nine months and then pods mummify (Fig 1f) [26].

Different methods could be utilized to control this disease. Chemical control has proved to be successful, but the use of chemical fungicides is still not optimized due to the lack of biological and epidemiological information on the pathogen. Periodic removing of the diseased pods from the fields is effective but is very time and labor consuming. Genetic resistance has the potential to be the best approach for long-term and cost-effective control. CATIE-R6 is an example of a genotype that exhibits a high incomplete resistance to MPR, with less than 10% of disease incidence over past 10 years [27]. However, incomplete resistance can vary according to weather conditions. In addition, there is a small number of resistant varieties that do not met the demand by the existing clonal propagation methods [2]. MPR control methods has limited success, being rarely accessible to smallholders, and also due to the limited knowledge on the pathogen biology and disease epidemiology that could help optimize these methods.

Pod infection success is determined by several factors, including pod wetness. It has been reported that the presence of a film of water over the pod is required for spore germination [23, 28–31]. High relative humidity (80 to 100%) and warm temperatures (20 to 27°C) are suitable conditions for spore germination and for the fungal penetration [26, 32, 33]. Favorable conditions for sporulation are similar to those required for infection. Sporulation is also dependent on fruit moisture and warm temperatures (20 to 28°C) but this last factor must fluctuate in order to stimulate the sporulation process [31, 34]. Light is also another important factor in the sporulation process. Alternate periods of light and darkness promote the spore formation *in vitro* [35].

Weather influence over MPR has been reported by several authors, mostly during the seventies and eighties. Barros Nieves [36] and Phillips [32] have concluded that high relative humidity due to excess of shade and poor ventilation within the plantation favors the frequency and intensity of the attack. Merchán [37] has established a positive correlation between the MPR incidence and the relative humidity at 60 days before symptom appearance. In addition, Torres de la Cruz, Ortiz García [38] found that MPR incidence is positively associated with periods of relative humidity higher than 90% recorded during the 49 days before symptom appearance. Rainfall has been also studied. Evans [39] and Porras and González [40] have reported a positive correlation between disease incidence and the amount of rainfall two to four months before the infection. Also, Maddison, Macías [41] concluded that in regions with a well-defined dry season, disease incidence tends to decrease as rain ceases, particularly if flowering decline. Another studied factor is temperature. Torres de la Cruz, Ortiz García [38] found that MPR incidence increased when temperatures ranged from 20 to 27°C, 49 days before symptom appearance. Finally, Suárez [25] has stated that a temperature daily range of 22 to 32°C favors MPR incidence and that cooler temperatures cause less severe attacks as incubation periods become longer. Available information of the weather influence over the disease is still scarce and outdated. Epidemiological field studies are almost inexistent, since the existing studies mostly consist of linear regressions between disease incidence and a single variable in a short period.

Today, new statistical tools are available that are increasingly used in phytopathology and other agronomic disciplines [42, 43]. For instance, Bugaud, Joannès-Dumec [44] implemented logistic regression models to explore the effect of the pre-harvest temperature on the chilling susceptibility of banana fruits stored at 13°C and particularly to identify the fruit growth stage that is the most impacted by the chilling injury. Anco, Madden [45] used linear and non-linear models to fit their data in order to examine effects of temperature, wetness duration and interrupted wetness duration over the sporulation rate of *Phomopsis viticola* on infected grapes. Finally, in a cacao system, Ndoumbè-Nkeng, Efombagn [46] used cross-correlation and multiple-regression analyses to better understand the relation between the incidence of Phytophthora Pod Rot and two environmental factors: rainfall and temperature. To our knowledge this kind of approach has not been applied to MPR until now.

This study aims to apply advanced statistical modeling to establish the relationships between three microclimatic variables (temperature, relative humidity and wetness) and MPR development. The statistical method applied allows us to 1) to determine, with no *a priori* assumptions, the specific period of the pod life where each microclimatic variable has the greatest influence on disease development and then 2) study all the variables over the determined periods and their interactions. This research aims to contribute to filling the existing knowledge gaps concerning microclimatic effects on the MPR epidemic by proposing two models based on microclimatic variables to explain appearance of symptoms and sporulation on the pods.

## Materials and methods

### Experimental site

Study was conducted at the Experiment on disease tolerant clones or L6 trial located at La Lola experimental farm of the Tropical Agricultural Research and Higher Education Center (CATIE, Spanish acronym). The farm is located in 28 Millas, Bataán District, Matina Canton, Limón Province, in the humid and cloudy tropical forest [47]. La Lola is located at 40 m.a.s.l, 10°06' latitude North and 83°23' longitude West, on the Atlantic Coast of Costa Rica. Average rainfall (1949–2010) is 3,575mm with a decrease in March and September. September is the month with less rainfall. Monthly average temperature ranges (1952–2010) were between 20.5 and 30°C. May and June are the warmest months, whereas December and January are the coldest [27]. Relative humidity averaged above 91% in the same period. The prevailing climate is influenced by the Caribbean: humid with a not well-defined dry season, quite cloudy with few sunny hours. All these characteristics correspond to the ideal environment for MPR development.

The L6 experiment was planted in 1998 and 1999. Forty-two clones, selected primarily for their high resistance to disease and/or high productivity, were compared in a randomized complete block experimental design, with four replicates of eight trees each. Planting distance is 3 x 3 m. Permanent shade in this experiment is distributed unevenly and is composed by immortelle (poró, *Erythrina poepiggiana*) and guava (*Inga edulis*) trees. Cacao and shade trees receive periodic maintenance prunings [27].

From the 42 clones included in the L6 trial, we used the cacao clone Pound-7 in our study. Pound-7 was selected based on its high susceptibility (incidence = 86% 2008–2013 average) to MPR and high capacity of fruit production (to assure the presence of pods throughout the year) [27]. Its average production is 542 kg/ha/yr (historical data average of 11 years) but its production potential has been severely decreased because of the disease, since its incidence reaches 75%.

### Pod measurements

Every week, generations (cohorts) of emerging pods between 3 and 10 cm (1 to 2 months’ age) were tagged from 29 May 2012 (the first generation) until 12 June 2013 (the 55^th^ generation), cumulating 2268 pods, corresponding to the entire production of the studied trees. Pods were individually observed for MPR symptoms and signs every week, from week one to 10 after tagging, and every two weeks, after week 10. Different categories of tagged pods were monthly removed from the trees: 1) pods with MPR sporulated lesions, 2) mature healthy pods ready for harvest and 3) pods with symptoms and signs of other diseases (mainly Phytophthora pod rot).

We defined three phytosanitary pod types: 1) healthy, 2) with MPR lesions with no signs of sporulation and 3) with MPR sporulated lesions. We had two categories of phytosanitary status change: from healthy to diseased pods with no signs of sporulation (pictures from a to d only, in Fig 1), and from diseased with no signs of sporulation to pods with sporulated lesions (pictures e and f, in Fig 1). These categories as referred to as H→D change and D→S change in the rest of the manuscript, respectively. From the 11^th^ week (pod age of four to five months), pods were evaluated every two weeks, because symptom or sign appearance is not as fast as in younger pods.

### Microclimatic data recording and behavior during the evaluation period

Microclimatic data were recorded by a Hobo H21-001 weather station (Onset Computer Corporation, Bourne, MA, USA) positioned within one plot of the trial. A total of nine sensors were installed in the middle of the eight trees of a plot (CC-137, repetition four) at different heights: three for temperature (S-TMB-M006), two of them at 2 m from ground level and one at 1m; four for wetness (S-LWA-M003) at 1. 25 m and two for relative humidity and temperature (S-THB-M008) at 1.5 m. Having several sensors was important to capture the microclimate variability within the plot. Climatic data was recorded every 30 seconds and averaged every 15 minutes. Temperature and relative humidity sensors were previously calibrated and corrections were applied to homogenize the data. In addition, wetness sensors were field calibrated to determine the wet/dry transition point. This transition point was determined based on the values that recorded the weather station at the moment when the pods and vegetative tissue change from wet to dry in the early morning.

The software HOBOware^®^ Pro was used to collect the data from the data logger weekly. Rainfall was recorded by a rain gauge located near the study site.

The trial meteorological information is presented in S1 Fig. Wetness frequency and relative humidity presented the same behavior throughout the day. This pattern was inverted with the temperature daily pattern. The highest temperatures of the day were registered at noon. The coolest temperatures were recorded in the early morning, about 6:00 and 7:00. Average maximum temperature was almost 29°C and the lowest almost 22°C. All the wetness sensors were wet (frequency = 100%) at night and early morning, from 18:00 to 7:30. During daytime, on average, at least one sensor was wet. The moment with less wetness was found around 13:30. Relative humidity was always high: on average, above 85%.

Daily total rainfall went from 0 up to 115mm. As shown in S2 Fig, this factor did not present any pattern. Rainfall was well-distributed during the entire observation period. A dry or rainy season could not be clearly distinguished throughout the year. However, a decrease in rainfall was observed between the beginning of January and the end of February 2013, when daily rainfall did not exceed 15.2 mm.

**Fig 2:**
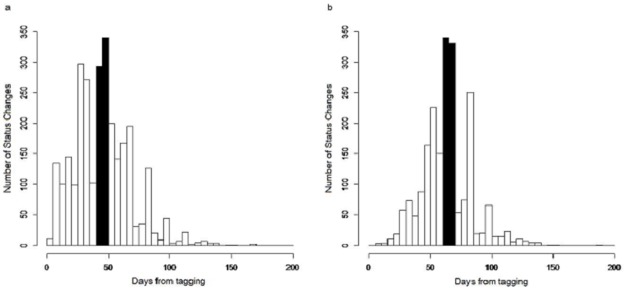
Histograms for the selection of the studied periods. a. Pod’s status change from healthy to diseased with no signs of sporulation. b. Pod’s status change from diseased with no signs of sporulation to sporulated lesions.

Our aim was to study the influence of the microclimatic variables on pod status changes. However, a short period after tagging, when changes occurred, needed to be defined to focus the analysis on pods of almost the same aged,having the same susceptibility level. We chose a 10-day period, for each of the two status change categories described earlier, when the major number of changes occurred, to enrich the statistical analyses. The response variable was the frequency or probability of change in this period. For H→D and D→S changes, we retained the period between 40 and 50 days after tagging (d.a.t.) and between 60 to 70 d.a.t., respectively (Fig 2). For the former, 22.4% of changes occurred during this period and for the latter, 32.2%.

Similar to what López-Bravo, Virginio-Filho [48] did, we calculated wetness average frequency based on the number of wet sensors among the four wetness sensors used. We preferred using the average frequency of wetness instead of wetness duration, because we considered it better reflects the real wetness condition in the heterogeneous environment of a cacao plantation.

### Data processing

For each pod and for each microclimatic variable, we integrated daily values (mean, sum or frequency, according to the nature of the variable) for all the possible periods between two dates relative to its tagging date (a starting date and duration). The starting date was considered up to 20 days before tagging and the duration of integration was done until the status change (H→D and D→S changes). We integrated daily values of *Wetness average frequency* (WF), *Average temperature* (Tmean), *Minimum temperature* (Tmin), *Maximum temperature* (Tmax), *Amplitude of temperature* (Tamp), *Minimum relative humidity* (RHmin) and *Total rainfall*. It was decided not to consider the daily *Maximum relative humidity* (RHmax) because its value at the study site was always 100%, as shown in S1 Fig. For this same reason, we did not consider the *Amplitude of Relative Humidity* (RHamp) or *Average Relative Humidity* (RHmean), since RHmin was the only variable source of variation.

### Statistical analyses

GLMM were used to explain the probability of status change of every observed pod as a function of microclimatic variables, using a binomial distribution (0 = healthy, 1 = diseased, for H→D changes; 0 = diseased, 1 = sporulated, for D→S changes). Microclimatic variables were included as fixed factors in models. We included the pod generation (i.e. the week it was tagged) as a random effect to account for the effect of the dynamic of the disease. All models were fitted with the glmer function in the lme4 package [49], in which the maximum likelihood of parameters is approximated by the Laplace method [50].

#### Single predictor GLMM analysis

**Fig 3:**
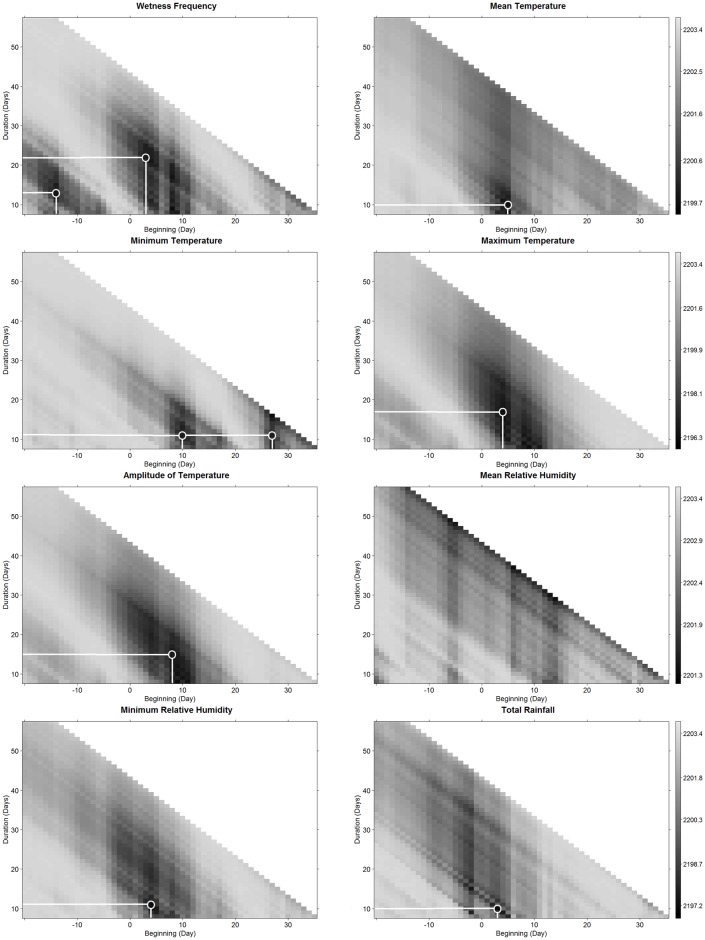
Period of influence of each daily variable on the pod status change, from healthy to diseased with no signs of sporulation, 40 to 50 days after tagging. By period of influence we meant from the starting day, with respect to tagging and duration from this day. The figure represents the AIC values of the binomial GLMMs explaining the pod’s status change from tagging, for each period of influence. On the starting date axis, zero corresponds to the tagging date of pods of 3 to10 cm in length. Circles indicate the lowest AIC value and the best microclimatic predictors of the pod status change (period of influence). The presence of a delimited surrounded black to gray zone indicates a zone of decreasing influence of the variable. Gray scale on the right represents the AIC values. Absence of circle indicates that no clear influence zone was identified.

**Fig 4:**
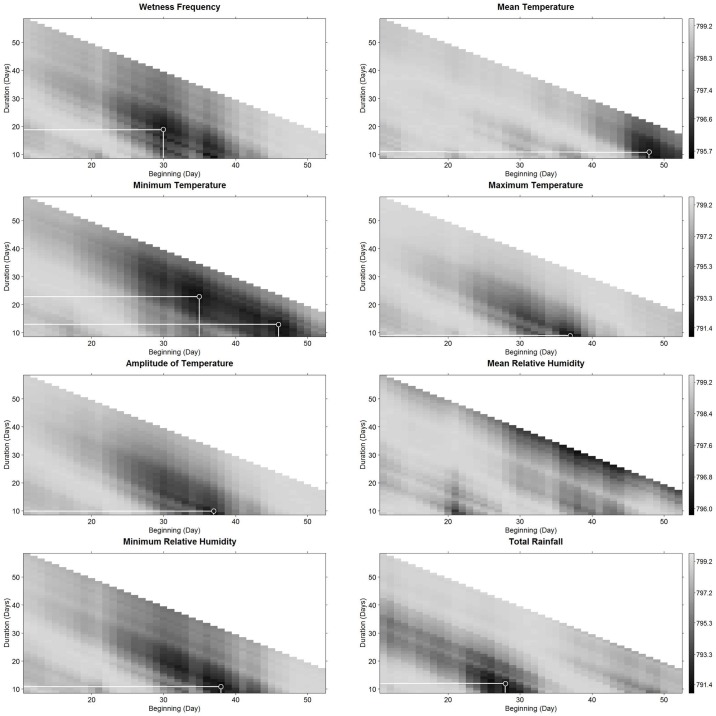
Period of influence of each daily variable on the pod status change, from diseased with no signs of sporulation to diseased with sporulated lesions, 60 to 70 days after tagging. By period of influence we meant from the starting day, with respect to tagging and duration from this day. The figure represents the AIC values of the binomial GLMMs explaining the pod status change from tagging, for each period of influence. On the starting date axis, zero corresponds to the tagging date of pods of 3 to10 cm in length. Circles indicate the lowest AIC value and the best microclimatic predictors of pod status change (period of influence). The presence of a delimited surrounded black to gray zone indicates a zone of decreasing influence of the variable. Gray scale on the right represents the AIC values. Absence of circle indicates that no clear influence zone was identified.

First we performed single variable GLMMs to determine the periods (starting date and duration) where each microclimatic variable better explained the two status changes (Figs 3 and 4). This was based on the calculation of the AIC value for each variable. The AIC is a criterion that measures the relative quality of a statistical model for a given data set [51]. The results of these comprehensive analyses of all possible periods of integration of each climatic variable were presented graphically in a grey scale, corresponding to the AIC value, according to each starting date and duration of integration. The centers of the areas with lowest AIC values were selected as most promising candidate variables for the second phase of the analysis. We then tested the correlation (Pearson coefficients) between candidate variables and kept those with a R^2^ < 0.9, also considering their potential effect on disease (based on literature). This step helped us to avoid having highly correlated microclimatic predictors of pod status change in the following complete GLMM analysis.

#### Complete GLMM analysis

The second step was to build two complete GLMMs (one model for H→D change and one for D→S change) including simultaneously all the candidate variables as fixed factors and *generation* (cohorts of pods produced each week) as random factors. The optimal model was obtained by using a backward model selection process (Drop1 function from R) to eliminate less-significant variables [52].

## Results

### Selection of the period of effect of each microclimatic variable

In the case of H→D change, the period of influence of almost all of the variables included the period from two to 24 d.a.t., except for wetness frequency (from the 14^th^ to one day before tagging, Table 1), and for minimum temperature (from 28 to 38 d.a.t., Table 1). In the case of D→S change, the period of influence of all the variables included the period from 30 to 60 d.a.t. (Table 2).

**Table 1:** Selected microclimatic predictors (starting date and duration) of pod status change from healthy to diseased with no sign of sporulation, from 40 to 50 days after tagging.

ID	Variables	Days *before* tagging	Days *after* tagging	Studied period	
**WF**_**-14 to -1**_	**Wetness frequency**	-20–18–16–**14–12–10–8–6–4–2**	0 2 4 6 8 10 12 14 16 18 20 22 24 26 28 30 32 34 36 38	40 42 44 46 48 50	52 54 56 58 60 62 64 66 68 70
**WF**_**3 to 25**_	**Wetness frequency**	-20–18–16–14–12–10–8–6–4–2	0 2 **4 6 8 10 12 14 16 18 20 22 24** 26 28 30 32 34 36 38	40 42 44 46 48 50	52 54 56 58 60 62 64 66 68 70
**Tmean**_**5 to 15**_	**Mean temperature**	-20–18–16–14–12–10–8–6–4–2	0 2 **4 6 8 10 12 14** 16 18 20 22 24 26 28 30 32 34 36 38	40 42 44 46 48 50	52 54 56 58 60 62 64 66 68 70
**Tmin**_**10 to 21**_	**Minimum temperature**	-20–18–16–14–12–10–8–6–4–2	0 2 4 6 8 **10 12 14 16 18 20** 22 24 26 28 30 32 34 36 38	40 42 44 46 48 50	52 54 56 58 60 62 64 66 68 70
**Tmin**_**27 to 38**_	**Minimum temperature**	-20–18–16–14–12–10–8–6–4–2	0 2 4 6 8 10 12 14 16 18 20 22 24 26 **28 30 32 34 36 38**	40 42 44 46 48 50	52 54 56 58 60 62 64 66 68 70
**Tmax**_**4 to 21**_	**Maximum temperature**	-20–18–16–14–12–10–8–6–4–2	0 2 **4 6 8 10 12 14 16 18** **20** 22 24 26 28 30 32 34 36 38	40 42 44 46 48 50	52 54 56 58 60 62 64 66 68 70
**Tamp**_**8 to 23**_	**Amplitude of temperature**	-20–18–16–14–12–10–8–6–4–2	0 2 4 6 **8 10 12 14 16 18 20 22** 24 26 28 30 32 34 36 38	40 42 44 46 48 50	52 54 56 58 60 62 64 66 68 70
**RHmin**_**4 to 15**_	**Minimum Relative Humidity**	-20–18–16–14–12–10–8–6–4–2	0 2 **4 6 8 10 12 14** 16 18 20 22 24 26 28 30 32 34 36 38	40 42 44 46 48 50	52 54 56 58 60 62 64 66 68 70
**TR**_**3 to 13**_	**Total Rainfall**	-20–18–16–14–12–10–8–6–4–2	0 2 **4 6 8 10 12** 14 16 18 20 22 24 26 28 30 32 34 36 38	40 42 44 46 48 50	52 54 56 58 60 62 64 66 68 70

**Table 2:** Selected microclimatic predictors (starting date and duration) of pod status change from diseased with no sign of sporulation to diseased with sporulated lesions, from 60 to 70 days after tagging.

ID	Variables	Days *before* tagging	Days *after* tagging	Studied period
**WF**_**30 to 49**_	**Wetness frequency**	-20–18–16–14–12–10–8–6–4–2	0 2 4 6 8 10 12 14 16 18 20 22 24 26 28 **30 32 34 36 38 40 42 44 46 48** 50 52 54 56 58	60 62 64 66 68 70
**Tmean**_**48 to 59**_	**Mean temperature**	-20–18–16–14–12–10–8–6–4–2	0 2 4 6 8 10 12 14 16 18 20 22 24 26 28 30 32 34 36 38 40 42 44 46 **48 50 52 54 56 58**	60 62 64 66 68 70
**Tmin**_**35 to 58**_	**Minimum temperature**	-20–18–16–14–12–10–8–6–4–2	0 2 4 6 8 10 12 14 16 18 20 22 24 26 28 30 32 34 **36 38 40 42 44 46 48 50 52 54 56 58**	60 62 64 66 68 70
**Tmin**_**46 to 59**_	**Minimum temperature**	-20–18–16–14–12–10–8–6–4–2	0 2 4 6 8 10 12 14 16 18 20 22 24 26 28 30 32 34 36 38 40 42 44 **46 48 50 52 54 56 58**	60 62 64 66 68 70
**Tmax**_**37 to 46**_	**Maximum temperature**	-20–18–16–14–12–10–8–6–4–2	0 2 4 6 8 10 12 14 16 18 20 22 24 26 28 30 32 34 36 **38 40 42 44 46** 48 50 52 54 56 58	60 62 64 66 68 70
**Tamp**_**37 to 47**_	**Amplitude of temperatura**	-20–18–16–14–12–10–8–6–4–2	0 2 4 6 8 10 12 14 16 18 20 22 24 26 28 30 32 34 36 **38 40 42 44 46 48** 50 52 54 56 58	60 62 64 66 68 70
**RHmin**_**38 to 49**_	**Minimum Relative Humidity**	-20–18–16–14–12–10–8–6–4–2	0 2 4 6 8 10 12 14 16 18 20 22 24 26 28 30 32 34 36 **38 40 42 44 46 48** 50 52 54 56 58	60 62 64 66 68 70
**TR**_**28 to 40**_	**Total Rainfall**	-20–18–16–14–12–10–8–6–4–2	0 2 4 6 8 10 12 14 16 18 20 22 24 26 **28 30 32 34 36 38 40** 42 44 46 48 50 52 54 56 58	60 62 64 66 68 70

Based on the results of a pair correlation analysis presented in S3 and S4 Figs, we discarded *Wetness Frequency* from three to 25 d.a.t. (WF_3 to 25_) and *Amplitude of Temperature* from eight to 23 d.a.t. (Tamp_8 to 23_) for H→D change, and *Amplitude of Temperature* from 37 to 47 d.a.t. (Tamp _37 to 47_) for D→S change.

### Best fitted models construction

**Fig 5:**
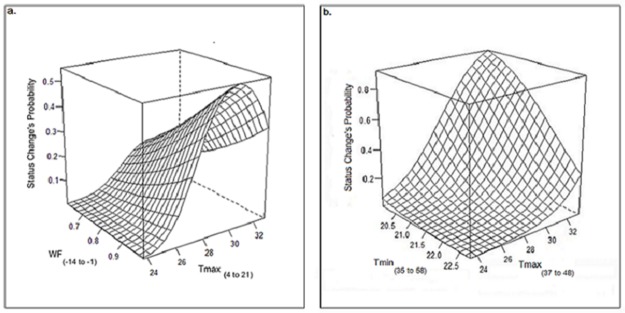
Best fitted models predictions. (a) Status change probability from healthy to diseased pod without sporulation between 40 to 50 days after tagging. (b) Status change probability from diseased pod without sporulation to diseased pod with sporulated lesions between 60 to 70 days after tagging **(**numbers between parentheses indicate the range of days of influence of each variable with respect to tagging).

After model selection WF_-14 to -1_, Tmax_4 to 21_ and the square of Tmax_4 to 21_ significantly predicted the H→D change (Table 3), and Tmin_35 to 58_ and Tmax_37 to 46_ the D→S change (Table 4). Model predictions of H→D change (Fig 5a) show that WF_-14 to -1_ had a positive relationship with the status change probability, except when Tmax_4 to 21_ was close to 24°C, when the probability of change was constantly null. With respect to Tmax_4 to 21_, there was a quadratic effect with a maximum at 30°C. However, the probability of change was low, with a maximum value of 0.55 predicted by the model. Model predictions of D→S change (Fig 5b) show that Tmin_35 to 58_ and Tmax_37 to 46_ are the most explanatory variables (microclimatic predictors) for explaining the probability of status change. Increasing temperature amplitude seemed to favor lesion sporulation. In our experiment, the highest change probability (0.9) was found for an average minimum temperature of 20°C (35 to 58 d.a.t.) and an average maximum temperature of 33°C (37 to 47 d.a.t.).

**Table 3:** Results of the analysis of deviance of the best fitted model for pod status change from healthy to diseased with no signs of sporulation, from 40 to 50 days after tagging.

Model	Df	AIC	logLik	deviance	χ2	χ2_Df	P
Complete model	5	2182.2	-1086.1	2172.2			
-WF_-14 to -1_	4	2186.8	-1089.4	2178.8	6.5338	1	0.01058
-Tmax_4 to 21_	4	2194.8	-1093.4	2186.8	14.541	1	0.0001372
-(Tmax_4 to 21_)^2^	4	2193.7	-1092.9	2185.7	13.476	1	0.0002417
Null model	2	2201.4	-1098.7	2197.4	25.145	3	1.44e^-05^

*Df* degrees of freedom, *AIC* Akaike

**Table 4:** Results of the analysis of deviance of the best fitted model for pod status change from diseased with no signs of sporulation to diseased with sporulated lesions, 60 to 70 days after tagging.

Model	Df	AIC	logLik	deviance	χ2	χ2_Df	P
Complete model	4	2197.1	-1094.6	2189.1			
Tmin_35 to 58_	3	2196.4	-1095.2	2190.4	1.2285	1	0.01058
Tmax_37 to 46_	3	2200.7	-1097.4	2194.7	5.6032	1	0.0001372
Null model	2	2201.4	-1098.7	2197.4	8.2662	2	0.01603

*Df* degrees of freedom, *AIC* Akaike

## Discussion

### Selected studied periods

Pound-7 is a highly susceptible clone to MPR. Symptoms were observed very early (27.9% in the first 30 d.a.t., and for this same period 4.4% of these pods were already sporulated). This has epidemiological and management implications. The removal of diseased pods, which is targeted toward the control of initial inoculum between pod generations (cohorts), should be accompanied with other control practices against secondary infection within the same pod generation, using protectant fungicides for instance.

However, the highest frequency of pod H→D change is from day 40 to 50 after tagging. This result is in accordance with previous reports indicating that appearance of symptoms occurs 40 to 60 days after infection [20]. Since pods were healthy but in the most susceptible stage at the starting moment, infection could occur right after tagging, i.e., when pods were about one or two months old [24]. According to Merchán [37], the most detectable symptom, the chocolate-colored spot, appears 75 days after infection and this lesion, as Phillips-Mora [6] has stated, will be covered with mycelium and spores within four to five days. This information also matches our results (Fig 4) for D→S change.

### Variables’ period of influence

Our analysis highlighted key variables, that affect the change status of pods in specific periods. These changes were observed in a regular, consistent and careful way, providing accurate information that supports the further conclusions of this work. The periods in which the preselected variables (included in the initial models) presented more influence for H→D change are between day three and day 25 after tagging, except for the wetness frequency. This means that during this period, the fungus is particularly active and its biological machinery and operation is very dependent on environmental conditions. Expression of symptoms appeared about 20 days after these variables produced their influence. For D→S change, the period of greatest influence is located between 28 to 58 d.a.t. This influence period is closer to the status change period (60 to 70 d.a.t.) than before (40 to 50 d.a.t) since the step between symptom appearance and sporulation is faster than between infection and symptom appearance, confirming that difference between the incubation and latency period is short [19].

### Pod status change: Healthy to diseased

#### Wetness and relative humidity effects

Wetness frequency from 14 to one day before tagging is one of the two variables that explain the probability of the H→D change. López [28], Chacín [29], Merchán [30], Campuzano [23] and Galindo [53] have stated that a film of water over the surface of the pod is necessary for germination to occur, since this factor stimulates the germ tube development. In conditions of high pressure of inoculum, germination and infection are then likely to happen in very early stages of the pod. As pods of 3 to10 cm in length were tagged, and assuming that they were between one and two months old, that means that we tagged apparently healthy pods that were already infected.

Relative humidity, despite being related to wetness frequency, did not appear to explain any status change since the experimental site is highly humid and the relative humidity variation is very low. On average, daily relative humidity was above 85%. Scherm and Van Bruggen [54] have observed a similar situation studying the effect of the fluctuating temperatures on the latent period of lettuce downy mildew. In their experiment, they focused on the temperature since humidity and light were not limiting for the disease growth, but the authors considered that these factors may have had a stronger effect than temperature on the colonization and the latent period of the disease. Similarly, for MPR, relative humidity could have had a higher effect than observed, under conditions of larger variation of this variable. Our results are in accordance to those obtained by Phillips [32], who demonstrated in pod bagging field experiments the importance of water film on the surface of the pods to allow the formation of the germ tube and to achieve penetration of the pathogen. It is reported that a relative humidity above 80% is optimal for fungus germination and growth [23, 55]. These wetness and humidity conditions were normally achieved every day during our experiment.

#### Temperature effect

Maximum temperature was the only temperature variable retained in the model. Similarly to relative humidity, variation of minimum temperature was probably too narrow in the study location (21.7 ± 1.3°C) to explain the probability of pod status change. Maximum temperature, however, had larger variation (30.3 ± 2.7°C), and in some cases could reach detrimental values. Maximum temperature period of influence happened late: four to 20 days after tagging. This variable is likely more important after penetration, for the intra- and intercellular colonization of the pod by the pathogen and thus for the expression of the symptoms [25]. We observed an optimum for this variable at approximately 30°C. Herrera [34] reported that the ideal range for growth and sporulation of the colonies in culture medium V8 is 24 to 28°C, at constant temperatures. This seems compatible with our results in field conditions. Cool or high temperatures seem to be harmful for the pathogen and inhibit the normal fungal development. While, to our knowledge, there are no reports on high maximum temperature effects on MPR in the field, there are indications that low temperatures and particularly low maximum temperatures are harmful to the disease. For instance, it is known that as altitude increases, the incidence of this disease goes down. Constant temperatures lower than 18°C severely limit the growth and sporulation of the pathogen *in vitro* [6].

### Pod status change: Diseased to sporulated

#### Temperature effects

As known, conditions that favor germination and penetration of *M*. *roreri* are different from those that favor production and release of the inoculum [32]. According to Hawker [56], the optimum temperature range for fungal sporulation is always lower than the optimum temperature range for its growth.

According to our results, sporulation process is determined by the daily temperature amplitude: large temperature amplitudes increase the probability for lesions to sporulate. We hypothesized that the lower temperatures could promote the spore formation, based on the fact that the presence of water is needed for germination, but it must be probed. On the other hand, the higher temperatures could shorten the period between the first symptoms (humps) and the sporulation. This means that temperature could affect in different ways, according to the fungal process. Tomerlin, Eversmeyer [57] found a similar behavior when studying the effect of the temperature on the development of brown rust on wheat under controlled conditions. These authors have reported that temperature has distinct effects on the different disease stages: latent and infectious periods. In their case, warmer temperatures shortened the latent period but also the infectious period. According to these results, warmer temperatures promote the beginning of sporulation but are detrimental to the continual spore formation. On the other hand, MPR is known to have a very long infectious period that could reach several months, since it could produce different sporulation cycles over the same infected tissue [26]. Therefore, MPR has two convenient strategies: 1) short period between appearance of symptoms and spore production and 2) long infectious period. This double strategy makes the disease very difficult to control.

In addition, high thermal amplitudes are more likely to happen in periods with a low number of rainy days, which normally buffer temperatures [48]. Sporulation is then promoted in periods when wind dispersal is also feasible. Spores of *M*. *roreri* are disseminated by a wind-dependent passive mechanism. In order for the spores to be liberated, low relative humidity is required. Without moisture, spores weigh less and could be easily removed from the pods by the air currents [40, 58, 59].

While for most pathogens, relative humidity is determinant for the sporulation process, this variable did not appear in our results because relative humidity was not a limiting factor in the experimental location. We think that the temperature effect over the sporulation represents valid knowledge to enrich a future epidemiological model. Lalancette, Foster [60] have mentioned that, sporulation models for predictive purposes are more efficient when the capacity of spores to be disseminated to new susceptible tissue and the infection potential are high, which is the case for MPR.

### Comparing status change probabilities between healthy-to-diseased and diseased-to-sporulated lesions

Maximum probability value for the H→D change (0.55) is considerably lower than the maximum value predicted for D→S change (0.90). This could be considered understandable since lesions of the diseased pods will likely produce spores whereas healthy pods will not all become diseased. As a consequence, the prediction of new diseased pods is more difficult than the prediction of sporulation knowing that pods are already infected. The low probability value exhibited by the final model for H→D change indicates that the model is more efficient for predicting unsuitable infection periods than propitious ones.

### Methodological approach

Our results constitute a good basis for improving already existing conceptual models, filling some existing knowledge gaps of the microclimatic effects over MPR epidemic. One of the existing conceptual model is the one developed by Leach, Mumford [61]. This model is based on management and economics field dynamics and aims to evaluate net returns of different management strategies for Central American farmers, but it presents understandable limitations, due to the lack of information on this disease. The incorporation of the microclimatic variables into conceptual models requires a precise understanding of the influence of these variables. The strength of our approach consists in the fact that there was no *a priori* on the influential period of each climatic variable on status-change probability. This means that the identification of the influential period is a result of our analysis and not a preset period established subjectively. Such an approach has recently been used in bananas, to study the influence of the temperature on the development of chilling injury during fruit growth [44]. It has been also used by Carval, Cotté [62] to study the effect of rainfall, temperature and biotic variables on the abundance of adult thrips on banana plants. This statistical approach is applied for the first time for fungal epidemiological studies. It allows analyzing every pod separately and not a proportion of incidence inside the population, offering more robust results and elevating the accuracy and power of the method.

## Supporting information

S1 Fig Meteorological mean values throughout the day (means of 462 days from 8 May, 2012 to 13 August, 2013). (TIF)

S2 Fig Daily rainfall distribution throughout the experimental period. (TIF)

S3 Fig Absolute values of Pearson’s correlation coefficients (r) within selected microclimatic variables for the status change healthy to diseased without sporulation. The larger the font size, the higher the correlation coefficient.
(TIF)

S4 Fig Absolute values of Pearson’s correlation coefficients (r) within selected microclimatic variables for the status change diseased without sporulation to sporulation. The larger the font size, the higher the correlation coefficient.
(TIF)

S1 Table Pod observations dataset. (XLS)

S2 Table Microclimate records dataset. (XLSX)
