# Supplementary figures and images for: Effects of microclimatic variables on the symptoms and signs onset of Moniliophthora roreri, causal agent of Moniliophthora pod rot in cacao
Source: PLoS One. 2017 Oct 3;12(10):e0184638. doi: 10.1371/journal.pone.0184638 (PMC5626025; doi:10.1371/journal.pone.0184638)

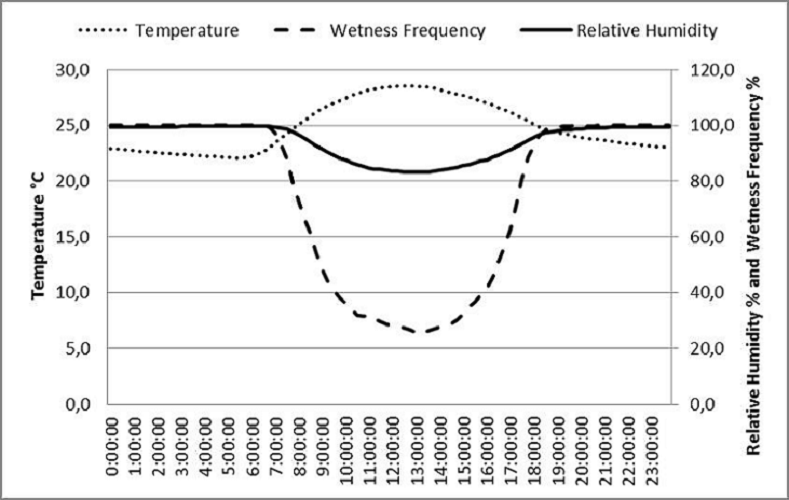

Supplement: S1 Fig — (TIF) [file pone.0184638.s001.tif]

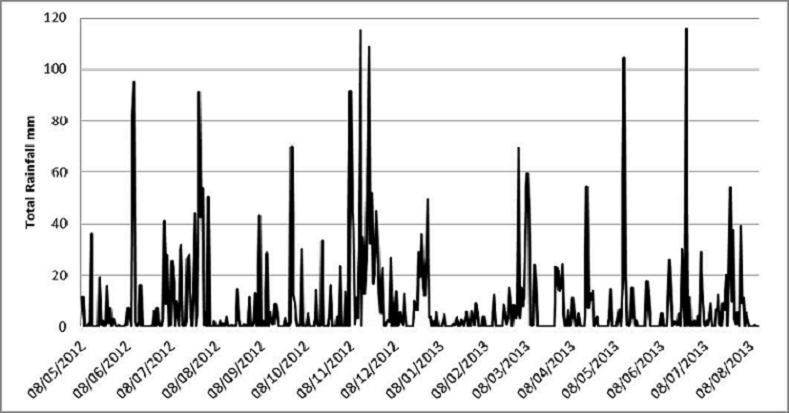

Supplement: S2 Fig — (TIF) [file pone.0184638.s002.tif]

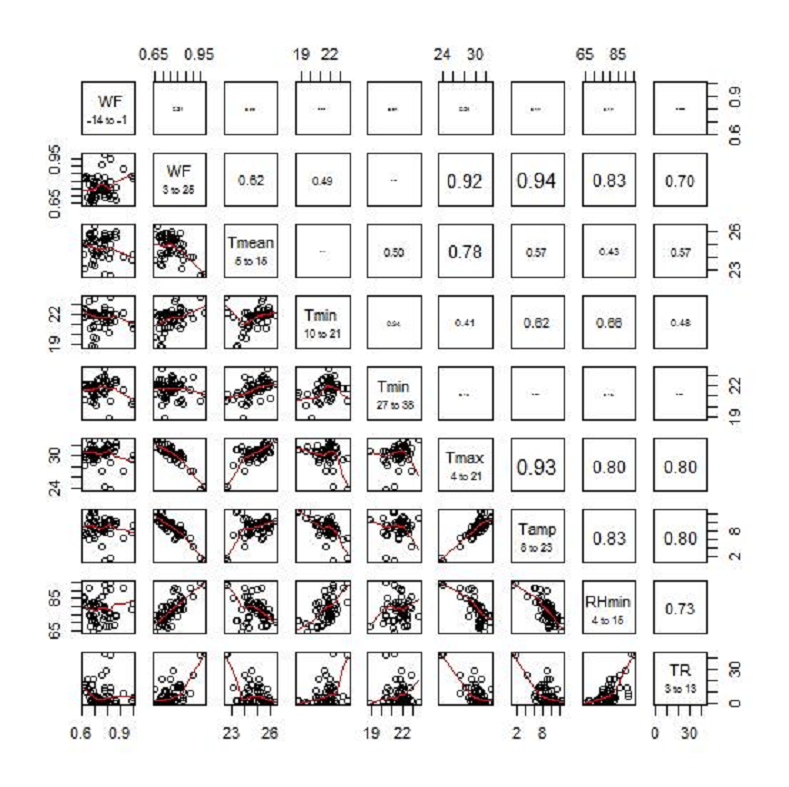

Supplement: S3 Fig — The larger the font size, the higher the correlation coefficient. (TIF) [file pone.0184638.s003.tif]

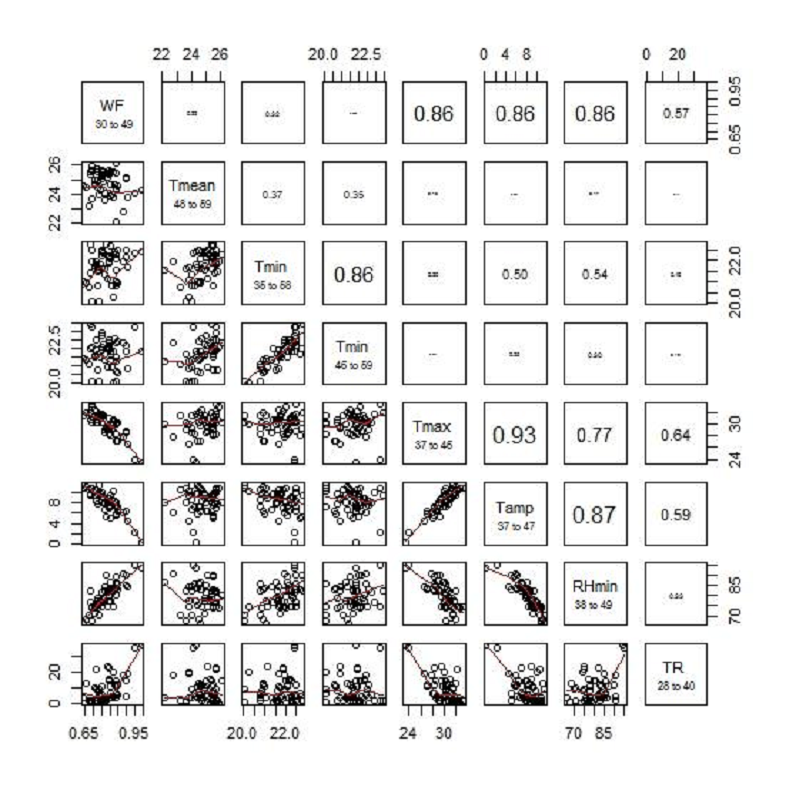

Supplement: S4 Fig — The larger the font size, the higher the correlation coefficient. (TIF) [file pone.0184638.s004.tif]
